# Role of characteristic impedance in carotid stiffness and cognitive dysfunction: interaction with proximal aortic stiffness

**DOI:** 10.1038/s41440-026-02650-4

**Published:** 2026-04-27

**Authors:** Chao-Feng Liao, Hao-Min Cheng, Chen-Huan Chen, Shao-Yuan Chuang

**Affiliations:** 1 National Yang Ming Chiao Tung University Hospital, Yilan, Taiwan, ROC; 2 Department of Medicine, National Yang Ming Chiao Tung University College of Medicine, Taipei, Taiwan, ROC; 3 Division of Faculty Development, Taipei Veterans General Hospital, Taipei, Taiwan, ROC; 4 Department of Medical Education, Taipei Veterans General Hospital, Taipei, Taiwan, ROC; 5 Institute of Public Health and Cardiovascular Research Center, National Yang Ming Chiao Tung University College of Medicine, Taipei, Taiwan, ROC; 6 Institute of Population Health Science, National Health Research Institute, Miaoli, Taiwan, ROC

**Keywords:** Characteristic impedance, Carotid stiffness, Aortic stiffness, Cognitive function

## Abstract

Increased stiffness in the proximal aorta and carotid artery, both crucial for regulating blood pressure and flow pulsatility, may contribute to cerebral microcirculation damage and cognitive decline. While aortic stiffness measured by aortic characteristic impedance (Zc) has been linked to suspected mild cognitive impairment (MCI), the role of carotid stiffness remains unclear due to inconsistent findings using traditional distensibility measures. This study investigates the relationship between carotid characteristic impedance (CCI) and suspected MCI, and examines how CCI interacts with Zc in contributing to cognitive dysfunction. A total of 1423 healthy community residents (average age 59.8 ± 11.7 years; 46.9% male) underwent comprehensive hemodynamic evaluations and carotid ultrasonography. CCI and Zc were calculated in the time domain, and the characteristic impedance ratio (CIR), defined as CCI/Zc, was used to assess the influence of impedance mismatch. Suspected MCI was determined using education-adjusted Mini-Mental State Examination (MMSE) cut-offs. Among participants, 478 (33.6%) were identified with suspected MCI. These individuals showed significantly higher CCI, while other carotid distensibility parameters were not significantly different. CCI was the only carotid stiffness measure independently associated with suspected MCI (OR per SD: 1.18; 95% CI: 1.02–1.36). CIR was negatively associated with MCI (OR: 0.84; 95% CI: 0.73–0.95), suggesting that a mismatch in impedance contributes to cognitive decline. The combination of elevated Zc and CCI was the strongest predictor of suspected MCI (OR: 2.10; 95% CI: 1.47–2.98). These findings underscore CCI as a sensitive and valuable marker for assessing carotid stiffness in relation to cognitive dysfunction.

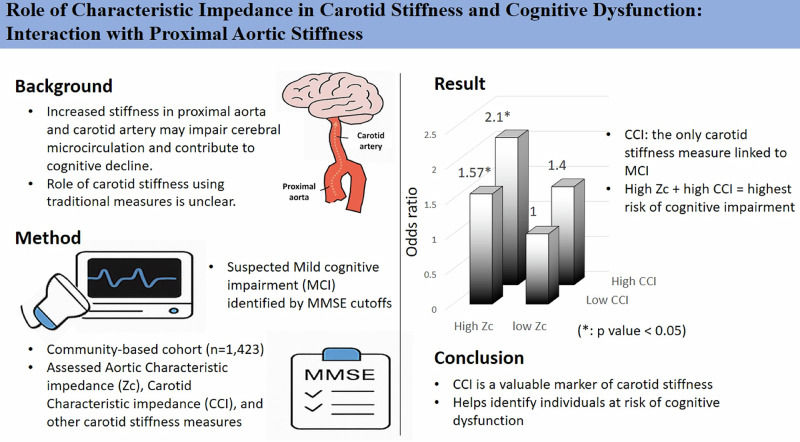

## Introduction

The future care needs and costs associated with age-related cognitive dysfunction and dementia have become a global concern due to population aging and increased life expectancy [1]. Given the absence of currently available curative therapies [2], early identification of cognitive impairment is crucial. It enables individuals to promptly access medical attention and preventive interventions.

Elastic vessels, such as the proximal aorta and carotid arteries, are crucial for buffering pulsatile blood flow and pressure [3] to maintain proper brain microcirculation. Arterial stiffness due to vascular aging would diminish this buffering capacity [4]. Furthermore, an impedance mismatch at the interface between the aorta and the carotid arteries, owing to differences in vessel characteristics and size [5], causes wave reflection and may serve as a barrier against excessive pulsatile flow into the brain [6]. A theory of “impedance matching” suggests that as vascular aging progresses, aortic impedance may “match” carotid impedance due to a greater increase in impedance in the former [6]. The “impedance matching” may disrupt the wave reflection interface. These phenomena lead to the excessive transmission of pulsatile energy into the brain, potentially disrupting microcirculation, impairing autoregulation, and damaging brain structures [7], ultimately contributing to brain injury and cognitive impairment [8].

In our previous study, aortic characteristic impedance (Zc), a surrogate measurement of proximal aorta stiffness, was found to be more sensitive than carotid-femoral pulse wave velocity (CFPWV) in individuals with suspected mild cognitive impairment (MCI) [9]. However, few studies have explored the relationship between carotid stiffness, assessed through distensibility parameters, and cognitive performance, and the findings have been inconsistent [10–12]. In contrast, characteristic impedance, which incorporates both mechanical arterial properties and geometry, has been shown to be a more sensitive indicator of stiffness in certain conditions [9, 13, 14]. Notably, no studies have examined the relationship between cognitive function and carotid stiffness using carotid characteristic impedance (CCI) as a surrogate marker. Therefore, this study aimed to investigate the association between CCI and cognitive dysfunction, as well as the interaction between Zc and CCI in relation to cognitive impairment, within the framework of “impedance matching.”

Point of view

- Clinical relevance
  Carotid characteristic impedance (CCI) showed a stronger association with cognitive function than conventional carotid stiffness measures, supporting its value as a better surrogate marker for carotid stiffness. This approach may improve the assessment of vascular contributions to cognitive decline.
- Future direction
  Future studies should adopt longitudinal designs to clarify the temporal and causal relationship between carotid stiffness and cognitive decline. In addition, the use of more sensitive and comprehensive neurocognitive assessments is warranted to better detect early and multidimensional cognitive changes.
- Consideration for the Asian population
  Increased pulsatile stress may accelerate vascular aging and cognitive decline, particularly in rapidly aging East Asian regions. CCI may provide additional value for risk stratification in this setting.

## Methods

### Study population

The participants in this study were recruited from the CardioVascular Disease Risk Factors Two-township Study (CVDFACTS) cohort through telephone outreach or mailed invitations. CVDFACTS is a continual community-based follow-up investigation centered on examining the pathophysiology and progression of risk factors associated with cardiovascular and cerebral diseases [15]. Two waves of recruitment were conducted, and further details have been described in our previous study [9]. A total of 1538 subjects were recruited, 1423 individuals completed all hemodynamic assessments and carotid ultrasonography examinations, rendering them eligible for inclusion in our analysis (Supplementary Fig. 1).

The study protocol details were outlined in our prior studies [9, 16]. In brief, participants underwent two separate visits spaced within 3 months. During the initial visit, we gathered baseline data including anthropometric measurements, blood chemistry, cognitive function assessments, and questionnaires. The second visit involved a thorough evaluation of cardiovascular hemodynamic assessments and carotid ultrasonography examination.

This study obtained approval from the Institutional Review Board of the National Health Research Institutes, Taiwan. Before enrollment, all participants provided written informed consent.

### Pulsatile hemodynamics assessment

In a quiet, temperature-controlled room, participants were positioned in a supine resting state following a minimum of 15 min of rest. Utilizing oscillometric techniques, a commercially available non-invasive vascular screening device (BP-203RPEIII, OMRON, Japan) was used to measure supine brachial systolic blood pressure (SBP) and diastolic blood pressure (DBP) over both arms. Pulse pressure (PP) was calculated as the difference between SBP and DBP. Applanation tonometry technique with a pencil-type tonometer incorporating a high-fidelity strain-gauge transducer in a 7-mm-diameter flat tip (SPC-350, Millar Instruments Inc, Texas) was applied for the recording of carotid pressure waveform [17].

The ensemble average wave of five to 10 consecutive carotid pressure waves was analyzed from the registered carotid waveforms. This ensemble average wave was then calibrated to brachial mean arterial pressure (MAP) and DBP [18]. MAP was computed as DBP plus one-third of PP. Carotid SBP was determined as the peak of the calibrated average carotid wave, and carotid PP was calculated as the difference between carotid SBP and DBP.

### Proximal aorta stiffness

#### Aortic characteristic impedance (Zc)

Zc was computed in the time domain using the calibrated right common carotid artery (CCA) pressure waveform as a surrogate for the aortic pressure waveform, given the minimal pressure amplification between the carotid artery and the aorta [19], Four consecutive beats of left ventricular outflow tract (LVOT) Doppler flows were manually traced using pulsed-wave Doppler echocardiography, resulting in a signal-averaged flow velocity waveform. The flow velocity was multiplied by the cross-sectional area of the LVOT to yield a volume flow waveform (Q _LVOT_). The onset and offset of LVOT flow were aligned with the foot and dicrotic notch of the carotid pressure waveform. The point where flow reached 95% of its peak value (ΔQ _LVOT_) and the corresponding change in pressure from the foot (ΔP) were identified. Zc was then estimated by calculating the ratio of the two values: Zc =ΔP /ΔQ _LVOT_ [14, 20].

### Carotid stiffness

#### Carotid characteristic impedance (CCI)

CCI were computed in the time domain. The bilateral common carotid flows were assessed using Doppler velocity waveforms obtained with a linear array probe featuring an imaging frequency range of 3.1–10.0 MHz. The probe was carefully positioned at the center of the CCA, approximately 1 cm beneath the carotid bulb. Subsequently, a series of 10 consecutive carotid flow waveform images was digitized and processed into a signal-averaged flow spectrum using the MATLAB program. The flow velocity was multiplied by the cross-sectional area of the CCA to yield a volume flow waveform (Q _carotid_). The point at which the volume flow waveform reaches 95% of its peak value (ΔQ _carotid_) and the concomitant change in pressure from the foot (ΔP) were identified. CCI was then estimated by calculating the ratio of the two values: CCI = ΔP /ΔQ _carotid_. The maximal value of CCI from both the left and right carotid arteries was utilized for subsequent hemodynamic analysis.

#### Carotid distensibility parameters

Carotid distensibility parameters were assessed and read with a commercialized vascular ultrasonographic device (Sonos 1000; Hewlett-Packard) by a single experienced technician to avoid inter-observer bias. All parameters were evaluated on both sides of CCA. Intima-media thickness (IMT) was estimated by the method modified from the standardized protocol of Howard et al. [21], and all measurements were done at the far wall of the CCA 1 cm beneath the bifurcation. The details of the measurement protocol were described in our previous study [22]. The value of IMT used in the present study was the maximal thickness of both sides. The carotid distensibility parameters can be constructed using the following equations [23]

$${Circumferential}\,{strain}\left({CS}\right)=\left(\varDelta D/D\right){\cdot }100\left( \% \right)$$

$${Distensibility}\,{Coefficient}\left({DC}\right)=\left(\varDelta A/A\right)/\varDelta P\\ =\left({\left(D+\varDelta D\right)}^{2}-{D}^{2}\right)/\left({PP}{{\cdot }}{D}^{2}\right)\left({10}^{-3}{kP}{a}^{-1}\right)$$

$${Compliance}\,{Coefficient}\left({CC}\right) =\varDelta A/\varDelta P\\ =\pi {{\cdot }}\left({\left(D+\varDelta D\right)}^{2}-{D}^{2}\right)/4{{\cdot }}{PP}\left({{mm}}^{2}{kP}{a}^{-1}\right)$$

$${Young}{{\hbox{'}}}s\,{elastic}\,{modulus}\left({YEM}\right)=D/\left({IMT}{{\cdot }}{DC}\right)\left({10}^{3}{kPa}\right)$$

$${Arterial}\,{stiffness}\,{index}\left(\beta \right):=\left[{Ln}\left({SBP}/{DBP}\right)\right]/\left(\varDelta D/D\right)$$

Where D and A are the end-diastolic carotid artery diameter and lumen area, and ΔD and ΔA are the stroke change of diameter and area, respectively.

CS and CC showed buffering capacity of the carotid artery, DC means the arterial stiffness of the carotid artery, YEM and *β* were the stiffness of the arterial wall material at applied pressure. Higher levels of YEM and *β* and lower levels of CS, CC and DC reflect greater arterial stiffness. Therefore, the maximal value of YEM and *β*, the minimal value of CS, CC and DC of both sides of CCA were presented in the study.

### Interaction between proximal aorta stiffness (Zc) and carotid stiffness (CCI) on cognitive dysfunction

We conducted subgroup analysis of different levels of Zc and CCI, and impedance variance between the proximal aorta and the carotid artery for further exploration of the interaction between Zc and CCI on cognitive dysfunction.

Both Zc and CCI were stratified into low and high groups based on their mean values (mean Zc: 96.7 dyne·s/cm^5^, mean CCI: 3138.1 dyne·s/cm^5^). Subsequently, subjects were categorized into four subgroups according to these levels.

Additionally, we used the characteristic impedance ratio (CIR), calculated as the ratio of carotid-to-aortic characteristic impedance (CCI/Zc), to quantify the impedance variance between the proximal aorta and the carotid artery. To ensure the suitability of this skewed variable for analysis, we applied a natural logarithm transformation to CIR to achieve normalization of its distribution. A higher CIR value indicates a greater disparity in impedance between these two vessels.

### Cognitive assessment

The assessment of global cognitive function was evaluated by the Chinese version of the Mini-Mental Short Examination (MMSE) [24]. Trained study nurses conducted face-to-face interviews at the study sites. The MMSE, which has a maximum score of 30 points, evaluates various cognitive aspects [24]. Since a single cut-point of MMSE was inadequate for diverse education levels in Asian ethnicities [25], we employed education-adjusted cut-points based on normative MMSE values in a large community-dwelling Chinese population to classify the subjects with suspected MCI [26] to improve sensitivity for detecting subtle cognitive decline in highly educated individuals. Given the known ceiling effect of the MMSE, particularly in populations with higher educational attainment. The cut-off scores were set as follows: <25 for individuals with no formal education, <27 for those with elementary school education, and <29 for those with junior school education or higher.

In addition, sensitivity analyses using the conventional MMSE cutoff of <26 were performed, and the results are presented in the Supplementary Material.

### Statistical methods

Continuous variables were described as means with standard deviation (SD), while categorical variables were expressed using numbers with percentages. Baseline characteristics, blood chemistry, hemodynamic parameters and indices of carotid stiffness were compared between normal and suspected MCI groups using t-tests for continuous variables and Chi-square tests for categorical variables. Linear regression was used to assess the associations between these variables and MMSE scores. Univariate and multivariable logistic regression analyses were employed to investigate the predictors of suspected MCI, presenting odds ratios (OR) per 1-SD increment and 95% confidence intervals (CIs). To better understand how age and suspected MCI affect carotid stiffness indices, subjects were divided into three age groups: under 50 years, between 50 and 70 years, and over 70 years. We conducted simple linear regression to examine the relationships between age and Zc, CCI, and CIR within each subgroup. All statistical analysis was performed using Statistical Package for SAS 9.4 (SAS Institute, Inc., Cary, NC).

## Results

### Characteristics of subjects with and without suspected MCI

A total of 1423 subjects (age 35 to 96 years old, average 59.8 ± 11.7 years, male 46.9%) completed all examinations and were eligible for further analysis. 478 subjects (33.6%) were categorized into the suspected MCI group according to the education-adjusted MMSE cut-points (Table 1). Subjects with suspected MCI exhibited significantly lower education levels (Table 1). Individuals, whether with or without suspected MCI, demonstrated similar age, sex distribution, prevalence of hypertension, diabetes mellitus and smoking, body mass index, renal function, lipid profile, MAP and HR (Table 1). Subjects with suspected MCI displayed significantly higher Zc, CCI and carotid PP compared to those without suspected MCI.

**Table 1:** Characteristics of the study population and groups of normal cognitive function and suspected MCI

	Total (*n* = 1423)	Normal (*n* = 945)	Suspected MCI (*n* = 478)	*P* value
Age	59.8 ± 11.8	59.3 ± 11.2	60.8 ± 12.8	0.064
Gender (male, %)	667(46.9)	445(47.1)	222(46.5)	0.830
Education levels (*n*, %)				**0.010**
No	19(1.3)	6(0.6)	13(2.7)	
Elementary school	225(15.8)	151(16.0)	74(15.5)	
Junior school	186(13.1)	104(11.0)	82(17.2)	
High school	455(32.0)	313(33.1)	142(29.7)	
University or higher	538(37.8)	371(39.3)	167(34.9)	
Hypertension (*n*, %)	442(31.1)	281(29.7)	161(33.7)	0.130
Diabetes mellitus (*n*, %)	210(14.8)	138(14.6)	72(15.1)	0.810
Smoking (*n*, %)				
non-smoker	1052(73.9)	697(73.8)	355(74.3)	0.860
previous smoker	227(16.0)	156(16.5)	71(14.9)	
current smoker	144(10.1)	92(9.7)	52(10.9)	
BMI	24.64 ± 3.49	24.63 ± 3.45	24.66 ± 3.57	0.946
Creatinine (mg/dL)	0.8 ± 0.36	0.79 ± 0.31	0.83 ± 0.45	0.229
Glucose (mg/dL)	101.28 ± 23.94	101.32 ± 24.17	101.21 ± 23.53	0.860
LDL (mg/dL)	117.53 ± 35.55	117.33 ± 35.20	117.93 ± 36.25	0.787
Triglyceride (mg/dL)	127.11 ± 80.79	126.97 ± 72.94	127.37 ± 94.46	0.340
MAP (mmHg)	91.91 ± 13.38	91.88 ± 13.07	91.97 ± 13.97	0.604
HR (beat/min)	67.13 ± 10.24	67.01 ± 10.23	67.36 ± 10.25	0.566
Carotid SBP (mmHg)	115.41 ± 16.71	115.03 ± 16.71	116.16 ± 16.71	0.288
Carotid PP (mmHg)	39.07 ± 9.91	38.64 ± 9.59	39.93 ± 10.47	**0.044**
Index of aortic stiffness				
Zc (dyne·s/cm^5^)	96.66 ± 39.18	94.25 ± 39.22	101.42 ± 38.7	**<0.001**
Indices of carotid stiffness				
CCI (dyne·s/cm^5^)	3138.1 ± 1262.78	3073.1 ± 1209.20	3266.7 ± 1354.67	**0.019**
CS (%)	5.84 ± 2.66	5.82 ± 2.57	5.88 ± 2.82	0.941
CC (mm^2^/kPa)	0.65 ± 0.31	0.65 ± 0.31	0.64 ± 0.32	0.422
DC (10^−3^/kPa)	24.42 ± 12.66	24.45 ± 11.95	24.36 ± 13.99	0.257
YEM (10^3^/kPa)	0.51 ± 0.36	0.49 ± 0.31	0.54 ± 0.43	0.375
β	8.45 ± 4.86	8.28 ± 4.56	8.80 ± 5.40	0.332
Diameter (mm)	6.3 ± 0.70	6.3 ± 0.70	6.3 ± 0.80	0.379
IMT (mm)	0.75 ± 0.38	0.75 ± 0.40	0.76 ± 0.32	0.266
CIR^a^	3.48 ± 0.49	3.49 ± 0.50	3.46 ± 0.47	0.402

*β* Arterial stiffness index, *CC* Compliance coefficient, *CCI* Carotid characteristic Impedance, *CIR* characteristic impedance ratio, *CS* Circumferential strain, *DC* Distensibility coefficient, *IMT* Intima-media thickness, *LDL* low-density lipoprotein-cholesterol, *MAP* mean arterial pressure, *MCI* mild cognitive impairment, *PP* pulse pressure, *SBP* systolic blood pressure, *YEM* Young’s elastic modulus, *Zc* aortic characteristic impedance

Numbers in bold letters indicate statistical significance

^a^transformed by using the natural logarithm

### Associations of indices of carotid stiffness with MMSE

In the univariate analysis, notable inverse correlations were observed between MMSE scores and variables such as CCI, YEM, and β, while significant positive correlations were found for CS, CC, and DC (Supplementary Table 1, Crude).

Upon adjusting for demographic factors, including age, sex, education levels, and MAP, and further controlling for comorbidities such as hypertension and diabetes mellitus, as well as lifestyle factors like smoking status, alongside physiological parameters like carotid artery diameter, height, and weight, the associations were refined. Only CCI, YEM, and β demonstrated a negative statistical significance in relation to MMSE scores. (Supplementary Table 1, Model 1 and 2).

### Associations of indices of carotid stiffness with suspected MCI

In the univariate analysis, several factors emerged as significant predictors for suspected MCI, including CCI (OR per 1-SD and 95% CI; 1.16, 1.04–1.29), and YEM (1.13, 1.02–1.26) (Table 2, Crude).

**Table 2:** Associations of indices of aortic and carotid stiffness with suspected MCI: logistic regression

	Crude		Model 1		Model 2	
	OR	95% CIs	OR	95% CIs	OR	95% CIs
**Indices of carotid stiffness**						
CCI	**1.16**	**(1.04–1.29)**	**1.19**	**(1.03–1.37)**	**1.18**	**(1.02–1.36)**
CS	1.02	(0.92–1.14)	1.05	(0.93–1.18)	1.06	(0.94–1.19)
CC	0.98	(0.87–1.09)	0.97	(0.85–1.10)	0.98	(0.86–1.12)
DC	0.99	(0.89–1.11)	0.99	(0.88–1.12)	1.05	(0.92–1.19)
YEM	**1.13**	**(1.02–1.26)**	1.05	(0.94–1.18)	1.11	(0.99–1.25)
β	1.11	(0.99–1.24)	1.07	(0.96–1.21)	1.07	(0.96–1.21)
IMT	1.06	(0.80–1.40)	0.97	(0.71–1.32)	0.97	(0.71–1.32)
**Index of aortic stiffness**						
Zc	**1.20**	**(1.07–1.34)**	**1.22**	**(1.08–1.37)**	**1.22**	**(1.08–1.37)**
**CIR**^a^	0.92	(0.82–1.03)	**0.84**	**(0.74–0.96)**	**0.84**	**(0.73–0.95)**

*β* Arterial stiffness index, *CC* Compliance coefficient, *CCI* Carotid characteristic Impedance, *CIR* characteristic impedance ratio, *CS* Circumferential strain, *DC* Distensibility coefficient, *IMT* Intima-media thickness, *YEM* Young’s elastic modulus, *Zc* aortic characteristic impedance

odds ratios (per SD increase, with corresponding 95% CIs), numbers in bold letters indicate statistical significance

Model 1: Adjusted age, gender, education levels, MAP, HR, carotid diameter, height, weight

Model 2: Adjusted age, gender, education levels, MAP, HR, carotid diameter, height, weight, hypertension, DM, LDL, Smoking

^a^transformed by using the natural logarithm

Upon conducting multivariable analysis and adjusting for key demographic and physiological variables such as age, sex, education levels, and MAP, it was revealed that CCI stood out as the only predictor significantly associated with suspected MCI (1.19, 1.03-1.37) (Table 2, Model 1). Further adjustment for additional factors such as heart rate, carotid artery diameter, height, weight, hypertension, diabetes mellitus, LDL cholesterol, and smoking status maintained the significance of CCI as the predictor for suspected MCI (1.18, 1.02-1.36) (Table 2, Model 2).

### Association of proximal aortic stiffness and carotid stiffness with MMSE and suspected MCI

**Fig. 1:**
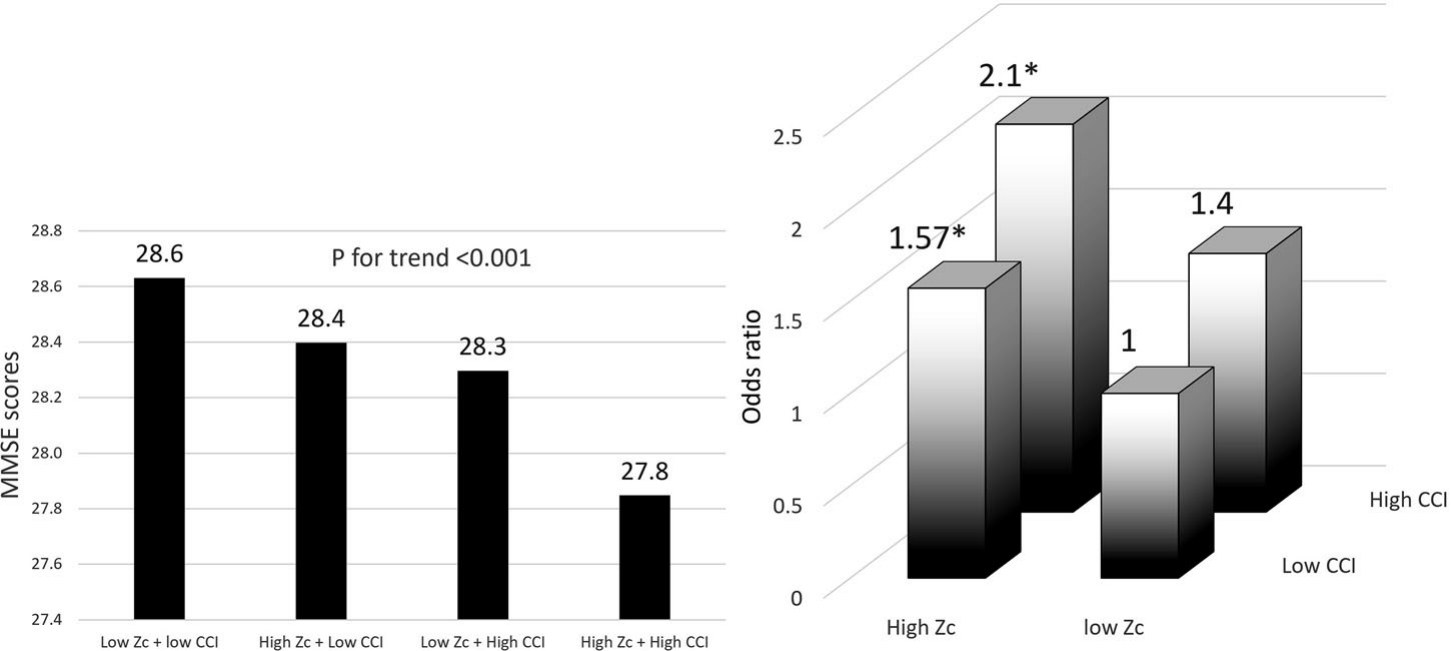
left panel: Effect of proximal aortic stiffness and carotid stiffness on MMSE. The estimated least square mean value was demonstrated after adjusting for age and sex. Right panel: Odds ratio of suspected MCI in subgroups (compared to the low Zc + low CCI group), *: *p* value < 0.05

Consistent with our previous findings, Zc remained a significant predictor of suspected MCI, even after adjusting for relevant covariates and confounding factors (Table 2). To further explore the impact of proximal aortic and carotid artery stiffness on cognitive function, subgroup analyses and impedance variance assessments were performed using the characteristic impedance of the proximal aorta (Zc) and carotid artery (CCI). Subgroups according to Zc and CCI levels demonstrated a discernible decline in MMSE scores with increasing stiffness in both the aortic and carotid regions (Fig. 1, left). Relative to the low Zc and low CCI group (*N* = 501, reference group), all three other subgroups exhibited elevated odds ratios for suspected MCI in the univariate models (Table 3, Crude). In the multivariable analysis, both the high Zc and low CCI group (*N* = 333) and the high Zc and high CCI group (*N* = 290) displayed significantly greater odds ratios for suspected MCI (odds ratio: 1.56, 95% CIs: 1.15–2.13, and odds ratio: 2.1, 95% CIs: 1.47–2.98, respectively) compared to the reference group (Table 3, Model 1 and 2) (Fig. 1, Right).

**Table 3:** Odds ratio of suspected MCI in subgroups (divided by Zc and CCI level)

	Crude		Model 1		Model 2	
	OR	95% CIs	OR	95% CIs	OR	95% CIs
Low Zc + low CCI(*N* = 501)	1		1		1	
High Zc + Low CCI(*N* = 333)	**1.47**	**(1.09–1.99)**	**1.56**	**(1.14–2.11)**	**1.56**	**(1.15–2.13)**
Low Zc + High CCI(*N* = 299)	**1.39**	**(1.02–1.89)**	**1.43**	**(1.02–2.01)**	1.40	(0.99–1.98)
High Zc + High CCI(*N* = 290)	**1.94**	**(1.43–2.64)**	**2.11**	**(1.49–2.99)**	**2.10**	**(1.47–2.98)**

*CCI* carotid characteristic impedance, *CIs* confidence interval, *OR* odds ratio, *Zc* Aortic characteristic impedance

odds ratios (per SD increase, with corresponding 95% CIs), numbers in bold letters indicate statistical significance

Model 1: adjusted for age, gender, education levels, MAP, carotid diameter, height, weight, HR

Model 2: adjusted for age, gender, education levels, MAP, carotid diameter, height, weight, HR, hypertension, DM, LDL, smoking status

We also analyzed the data using the conventional MMSE cutoff of <26, with the results presented in the Supplementary Material (Supplementary Table 2 and Supplementary Table 3). Participants classified as cognitively impaired by this cutoff were older, had lower educational attainment, a higher burden of comorbidities, and less favorable carotid stiffness parameters (Supplementary Table 2). In crude logistic regression, CCI showed the strongest association with lower MMSE ( < 26) (OR 1.81, 95% CI 1.55–2.13), and both carotid stiffness indices and Zc were also positively associated with lower MMSE (Zc: OR 1.28, 95% CI 1.08–1.50). After multivariable adjustment, these associations were attenuated and no longer statistically significant (Supplementary Table 3).

**Fig. 2:**
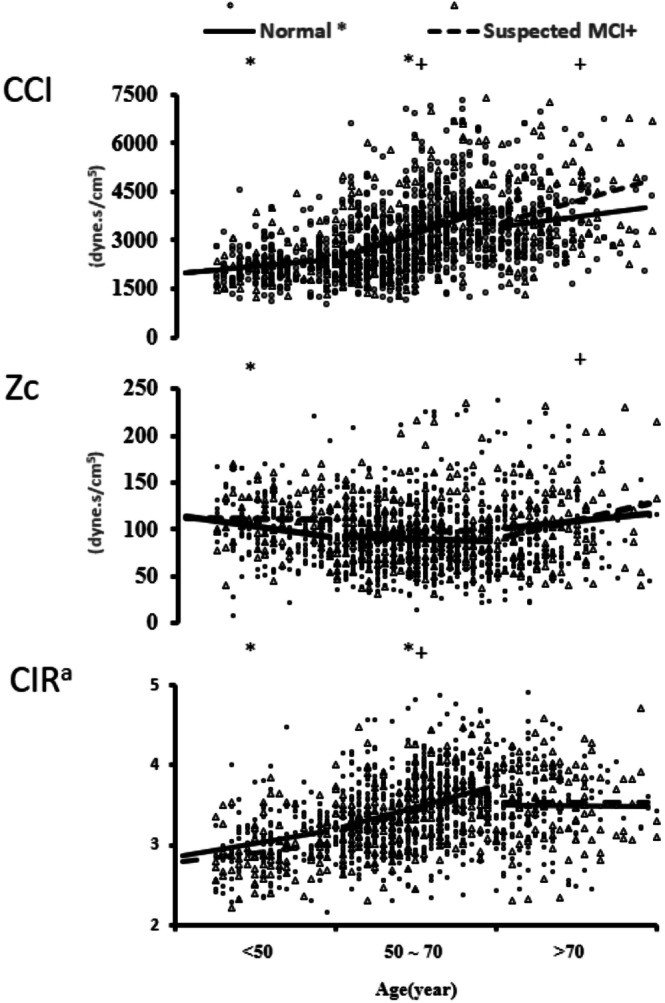
Scatter plot and linear regression line of CCI, Zc and CIR with age across the age groups in subjects with or without suspected MCI. *: the slope in normal subjects, *p* < 0.05, +: the slope in subjects with suspected MCI, *p* < 0.05, #: the difference in slopes between normal subjects and those with suspected MCI, *P* < 0.05. ^a^: transformed by using the natural logarithm

Although the CIR seemed similar between individuals with and without suspected MCI (Table 1), a greater CIR indicated a reduced risk of suspected MCI after adjustments for covariates and confounding factors (Table 2, Model 1 and 2). The linear regressions of CIR and its components Zc and CCI, based on age in subjects with and without suspected MCI, categorized into subgroups of <50 years, 50 to 70 years, and >70 years, were presented in Fig. 2. In individuals under the age of 50, we noted that CIR in the suspected MCI group remained relatively stable with aging. However, within the normal group, the increase in CIR with age primarily stemmed from a decline in Zc (Fig. 2). Conversely, among those aged between 50 and 70 years, we observed an elevation in CIR in both the normal and suspected MCI groups, driven by an increase in CCI (Fig. 2). Subsequently, among individuals over 70 years of age, CIR remained steady. Within the normal group, this stability was attributed to consistent Zc and CCI values, while in the suspected MCI group, both Zc and CCI exhibited age-related increases (Fig. 2).

We found notable correlations between Zc and suspected MCI in individuals under 50 years old and those between 50 and 70 years old (Table 4). Furthermore, CCI exhibited a significant link with suspected MCI solely among participants over 70 years old. Conversely, the CIR displayed a negative correlation solely in those under 50 years old. This phenomenon could be explained by the heightened Zc levels in the suspected MCI group within this age bracket, resulting in a diminished CIR.

**Table 4:** Associations of Zc, CCI and CIR with suspected MCI by age subgroups in logistic regression models

	Age <50 years		Age 50-70 years		Age >70 years	
	OR	95% CIs	OR	95% CIs	OR	95% CIs
Zc	**1.55**	**(1.10–2.20)**	**1.28**	**(1.08–1.51)**	0.94	(0.77–1.15)
CCI	0.93	(0.50–1.75)	1.19	(0.99–1.42)	**1.35**	**(1.01–1.82)**
CIR^a^	**0.65**	**(0.43–0.97)**	0.88	(0.74–1.05)	1.12	(0.85–1.47)

*CCI* carotid characteristic impedance, *CIs* confidence interval, *CIR* characteristic impedance ratio, *OR* odds ratio, *SD* standard deviation, *Zc* aortic characteristic impedance

Odds ratios (per SD increase, with corresponding 95% CIs), numbers in bold letters indicate statistical significance

Adjusted for age, gender, education levels, MAP, carotid diameter, height, weight, HR, hypertension, DM, LDL, smoking status

^a^transformed by using the natural logarithm

## Discussion

In our study, we explored the correlation between indices of carotid stiffness and suspected MCI in a healthy middle-aged to elderly cohort. Our results revealed that only CCI maintained its association with suspected MCI even after adjusting for various factors. This finding highlighted CCI as a pivotal carotid stiffness index for identifying cognitive function decline. Furthermore, the combined effect of elevated Zc and CCI proved to be the most sensitive predictor of suspected MCI, underscoring the critical role of conduit vessel stiffness in cognitive function.

Several previous studies have addressed the association between carotid stiffness and cognitive function. Research on former smokers with and without chronic obstructive pulmonary disease showed that carotid stiffness, measured by β, was associated with variability in executive function and processing speed [27]. Additionally, carotid stiffness, measured by carotid DC and YEM, was linked to lacunar infarction and white matter hyperintensity volume, potentially worsening cognitive function decline [28]. Three studies involving middle-aged populations identified that carotid stiffness, rather than aortic stiffness when using CFPWV as the surrogate measure, was significantly associated with cognitive function tests [11, 29, 30]. In contrast, the Rotterdam study, conducted in older individuals, didn’t establish an association between carotid DC, CFPWV, and cognitive function tests [10]. The Maastricht Study initially revealed an association between increased carotid stiffness, but not CFPWV, and poorer cognitive function [30]. However, it subsequently showed a positive association between CFPWV and MMSE scores, while no such association was observed with carotid distensibility [12]. The evidence linking cognitive function to direct measures of carotid stiffness remains inconclusive and is notably less robust compared to the evidence for systemic arterial stiffness [31]. In a previous study, the cerebral flow and stiffness indices were not independently associated with cognitive performance after multivariable adjustment in patients with degenerative aortic stenosis, while age, diabetes, renal dysfunction, and cardiac function emerged as the principal determinants of cognitive status [32]. These findings emphasize that stiffness markers are best interpreted as indicators of global cardiovascular risk rather than standalone determinants of cognitive impairment.

Compliance, distensibility, and elastic modulus are the most commonly used measures of carotid stiffness. Currently, there is no consensus on which measurement most accurately represents carotid stiffness. In the aforementioned studies, these parameters served as surrogates for carotid stiffness. Compliance measures the absolute volume change in response to local pressure changes, while distensibility reflects the relative volume change. The elastic modulus incorporates IMT to account for arterial wall thickness, providing a more comprehensive assessment of stiffness. However, these parameters were not associated with MMSE or suspected MCI after adjusting for covariates and potential confounders in the present study. In contrast, CCI, which combines mechanical arterial properties and geometrical features, offers a more comprehensive reflection of the dynamic relationship between arterial pressure and flow, thereby providing a more precise assessment of changes in carotid stiffness. In our data, the distensibility parameters were highly correlated with each other (Supplementary Table 4). However, despite the significant correlation between CCI and these distensibility parameters, the correlation coefficients were relatively low (Supplementary Table 4), suggesting that CCI represents a distinct and unique measure of carotid stiffness.

To the best of our knowledge, our study is the first to establish an independent association between CCI and suspected MCI. Furthermore, compared to other measures of carotid stiffness, the CCI proved to be the most sensitive and precise indicator of carotid stiffness in relation to cognitive dysfunction.

### Proximal aortic stiffness and carotid stiffness with cognitive dysfunction

The ARIC-PET study discovered that elevated heart-carotid PWV was most strongly linked to reduced brain volume, increased white matter hyperintensities, and greater amyloid-β accumulation than other aortic stiffness parameters [33]. Carotid stiffness was independently associated with amyloid-β accumulation in patients with amnestic mild cognitive impairment, whereas aortic stiffness, measured by CFPWV, did not show this association [34]. It may suggest that the excessive pulsatile damage transmitted from the heart to the brain microcirculation due to arterial stiffness was directly associated with the stiffness of connected conduit vessels. In our previous study, Zc, a surrogate measurement of proximal aorta stiffness, was found to be more sensitive than CFPWV, which primarily measures descending aorta stiffness, in individuals with cognitive dysfunction [9]. In the present study, we further examined the CCI, a surrogate measure of carotid stiffness, and found it to be strongly associated with cognitive decline. Moreover, the combined stiffness of the proximal aorta and carotid artery, which links the heart and brain, has emerged as a highly sensitive marker for cognitive dysfunction, underscoring the crucial role of conduit vessel stiffness in brain health.

In our study, we observed a negative correlation between CIR and suspected MCI, especially in individuals under 50 years old. A high CIR suggests a significant impedance mismatch at the aorta-CCA interface, potentially playing a protective role. However, contrary to the “impedance matching” theory, CIR increased rather than decreased with age, challenging its validity. As individuals age beyond 50 years, the purported protective effect of increasing impedance mismatch diminishes. Conversely, CCI increased after the age of 50 and emerged as the primary predictor of suspected MCI after the age of 70. The combination of high Zc and high CCI represented the most robust predictor. Therefore, the protective effect of impedance mismatch appears to work only when total impedance is low, and the buffering function is still intact in young adults, and the harmful “impedance matching” may play little role in the vascular aging-related cognitive function decline.

In our age-stratified analysis, cognitive decline in younger participants was more closely associated with proximal aortic stiffness, whereas carotid artery stiffness was more strongly related to cognitive impairment in individuals older than 70 years. This age-dependent pattern may reflect the temporal progression of vascular aging along the arterial tree.

Prior studies indicate that proximal aortic stiffening precedes that of peripheral elastic arteries, including the carotid artery. Increases in central aortic impedance have been shown to precede detectable changes in conventional arterial stiffness indices [13], while reduced ascending aortic strain and distensibility have been identified as the earliest manifestations of vascular aging [35]. This may relate to the high elastin content of the proximal aorta [5], which makes it susceptible to early elastin fragmentation and medial degeneration during aging and cardiometabolic stress. Early stiffening of the proximal aorta impairs its Windkessel function and facilitates transmission of pulsatile energy into the arterial circulation, including the cerebral microcirculation [36]. This may impair cerebral autoregulation and promote cerebral small-vessel disease [8]. In addition, it increased systolic and pulse pressures, thereby promoting arterial hypertension, a major risk factor for cerebral small-vessel disease and dementia. Consequently, proximal aortic stiffness parameters may better reflect early vascular changes associated with cognitive decline in younger individuals.

In contrast, stiffening of peripheral arteries such as the carotid artery tends to occur later in the vascular aging process. Carotid vascular changes become more pronounced with advancing age and accumulation of cardiovascular risk factors [37]. Recent evidence also suggests that carotid artery stiffness may be more strongly associated with cerebrovascular disease and cognitive impairment than aortic stiffness in older populations [38]. Therefore, carotid stiffness may better reflect the cumulative vascular burden affecting cerebral perfusion and microcirculation, which could explain its stronger association with cognitive decline in individuals older than 70 years.

## Strengths and limitations

Our study is the pioneering investigation to establish a link between cognitive function and carotid stiffness, utilizing CCI as the primary parameter. This novel approach enhances the understanding of vascular contributions to cognitive health. Through our research, we revealed that CCI exhibits superior sensitivity compared to other commonly employed parameters in similar studies. This underscores the effectiveness of CCI as a surrogate marker for carotid stiffness assessment.

The cross-sectional nature of our study impedes the establishment of a causal relationship between carotid stiffness and cognitive function decline. To address this limitation, future longitudinal studies are imperative to elucidate the temporal relationship between these variables. While the MMSE serves as a widely used tool for detecting cognitive impairment, its sensitivity to mild cognitive decline is restricted. To mitigate this limitation, we employed educated cut-off points for classifying subjects with suspected MCI. Nonetheless, the approach’s sensitivity and specificity were constrained, leading to unavoidable misclassifications. Despite this limitation, our findings consistently supported the association between carotid stiffness and cognitive function. We applied education-adjusted MMSE cutoff points rather than a single conventional threshold. Our aim was not to identify clinically overt cognitive impairment or mild cognitive impairment, but to capture subtle or early cognitive decline, particularly in a highly educated population. Previous studies have shown that the MMSE is subject to a ceiling effect in individuals with higher educational attainment, in whom small score reductions may still represent meaningful cognitive changes. In the present study, participants classified as cognitively impaired using the MMSE < 26 cutoff were notably older, had lower educational levels, and exhibited a higher burden of comorbidities, consistent with more advanced and overt cognitive dysfunction. Under this definition, the association between carotid stiffness and cognitive function was attenuated, reflecting the reduced sensitivity of the conventional MMSE cutoff for detecting subtle cognitive changes in cognitively normal, highly educated individuals.

## Perspective of Asia

The carotid artery serves as a key interface between the proximal aorta and the brain, delivering blood to the cerebral microcirculation while also transmitting potentially harmful pulsatile energy. In this study of approximately 1500 healthy Asian individuals, CCI was evaluated as a surrogate of carotid stiffness and showed a closer association with cognitive function. These findings suggest that stiffness-related pulsatile stress may contribute to cognitive decline. Given the growing burden of cognitive function decline in rapidly aging East Asian societies, the incorporation of impedance-based vascular markers such as CCI may improve risk stratification and prevention strategies.

## Conclusion

CCI showed a stronger association with cognitive function than conventional carotid stiffness measures. Moreover, the combination of proximal aortic stiffness and carotid stiffness emerged as a robust predictor of lower MMSE scores and suspected MCI. Our findings suggest that the stiffness of the proximal aorta and carotid artery has an additive effect on pulsatile damage in the brain’s microcirculation and cognitive function.

## Supplementary information

Supplementary materials
