# Supplementary material for: Role of characteristic impedance in carotid stiffness and cognitive dysfunction: interaction with proximal aortic stiffness
Source: Hypertens Res. 2026 Apr 27;49(7):2026–37. doi: 10.1038/s41440-026-02650-4 (PMC13333493; doi:10.1038/s41440-026-02650-4)
Supplement: Supplementary file 1 — Supplementary materials [file 41440_2026_2650_MOESM1_ESM.docx]

**SUPPLEMENTAL MATERIAL**

| 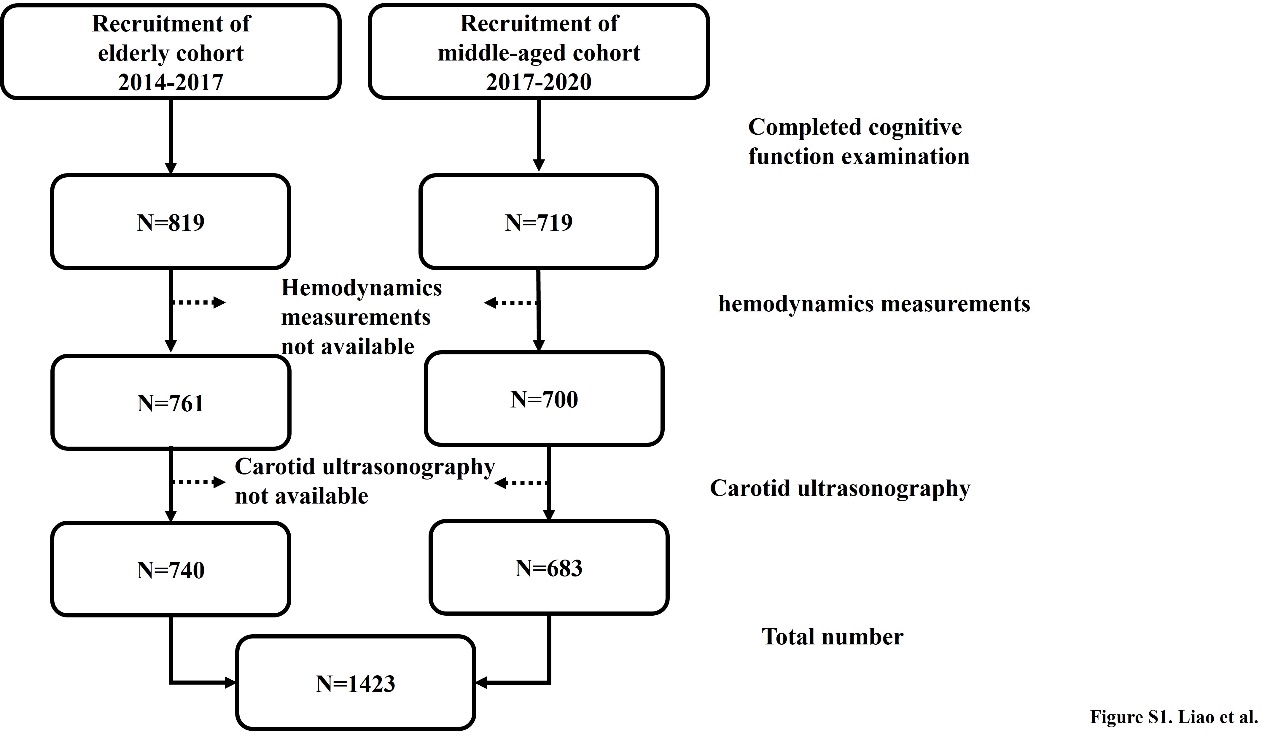  Supplementary Figure 1. flow chart of the study  Supplementary Table 1. The association between indices of carotid stiffness and MMSE | | | | | | |
| --- | --- | --- | --- | --- | --- | --- |
|  | Crude | | Model 1 | | Model 2 | |
|  | Standardized beta | P value | Standardized beta | P value | Standardized beta | P  value |
| CCI | **-0.210** | **<0.001** | **-0.083** | **0.007** | **-0.088** | **0.005** |
| CS | **0.065** | **0.016** | 0.024 | 0.354 | 0.022 | 0.410 |
| CC | **0.085** | **0.001** | 0.043 | 0.102 | 0.055 | 0.060 |
| DC | **0.121** | **<0.001** | 0.033 | 0.249 | 0.026 | 0.365 |
| YEM | **-0.148** | **<0.001** | **-0.093** | **<0.001** | **-0.089** | **0.001** |
| β | **-0.150** | **<0.001** | **-0.077** | **0.003** | **-0.074** | **0.005** |
| IMT | -0.052 | 0.050 | -0.007 | 0.784 | -0.005 | 0.857 |
| numbers in bold letters indicate statistical significance.  Model 1: Adjusted age, gender, education levels, MAP | | | | | | |
| Model 2: Adjusted age, gender, education levels, MAP, height, weight, hypertension, DM, LDL, Smoking, carotid artery diameter  β: Arterial stiffness index, CC: Compliance coefficient CCI: Carotid characteristic Impedance, CS: Circumferential strain, DC: Distensibility coefficient, IMT: Intima-media thickness, MMSE: Mini-Mental State Examination, YEM: Young’s elastic modulus | | | | | | |

| Supplementary Table 2. Characteristics of the study population and groups of normal cognitive function and MMSE<26 | | | | | | | |
| --- | --- | --- | --- | --- | --- | --- | --- |
|  | Total  (n=1423) | | Normal  (n=1311) | | MMSE<26  (n=112) | | P value |
| Age | 59.8 | ±11.8 | 58.9 | ±11.3 | 70.2 | ±11.6 | **<0.001** |
| Gender (male, %) | 667 | (46.9) | 626 | (47.9) | 41 | (36.9) | **0.028** |
| Education levels (n, %) | | |  |  |  |  | **<0.001** |
| No | 19 | (1.3) | 2 | (0.2) | 17 | (15.2) |  |
| Elementary school | 225 | (15.8) | 174 | (13.3) | 51 | (45.5) |  |
| Junior school | 186 | (13.1) | 171 | (13.0) | 15 | (13.4) |  |
| High school | 455 | (32.0) | 437 | (33.3) | 18 | (16.1) |  |
| University or higher | 538 | (37.8) | 527 | (40.2) | 11 | (9.8) |  |
| Hypertension (n, %) | 442 | (31.1) | 383 | (29.2) | 59 | (52.7) | **<0.001** |
| Diabetes mellitus (n, %) | 210 | (14.8) | 185 | (14.1) | 25 | (22.3) | **0.026** |
| Smoking (n, %) | |  |  |  |  |  | **0.038** |
| non-smoker | 1052 | (73.9) | 965 | (73.6) | 87 | (77.7) |  |
| previous smoker | 227 | (16.0) | 209 | (15.9) | 18 | (16.1) |  |
| current smoker | 144 | (10.1) | 137 | (10.5) | 7 | (6.3) |  |
| BMI | 24.64 | ±3.49 | 24.56 | ±3.49 | 25.61 | ±3.38 | **0.002** |
| Creatinine (mg/dL) | 0.8 | ±0.36 | 0.8 | ±0.37 | 0.85 | ±0.28 | 0.067 |
| Glucose (mg/dL) | 101.28 | ±23.94 | 100.83 | ±23.11 | 106.41 | ±31.63 | 0.232 |
| LDL (mg/dL) | 117.53 | ±35.55 | 117.74 | ±35.68 | 115.12 | ±34.04 | 0.557 |
| Triglyceride (mg/dL) | 127.11 | ±80.79 | 127.28 | ±82.17 | 125.11 | ±62.82 | 0.902 |
| MAP (mmHg) | 91.91 | ±13.38 | 91.59 | ±13.41 | 95.62 | ±12.48 | **0.002** |
| HR (beat/min) | 67.13 | ±10.24 | 67.26 | ±10.27 | 65.57 | ±9.81 | 0.089 |
| Carotid SBP (mmHg) | 115.41 | ±16.71 | 114.63 | ±16.35 | 124.5 | ±18.21 | **<0.001** |
| Carotid PP (mmHg) | 39.07 | ±9.91 | 38.41 | ±9.23 | 46.86 | ±13.62 | **<0.001** |
| Index of aortic stiffness |  |  |  |  |  |  |  |
| Zc (dyne·s/cm^5^) | 96.66 | ±39.18 | 95.74 | ±37.76 | 107.23 | ±51.97 | 0.109 |
| Indices of carotid stiffness |  |  |  |  |  |  |  |
| CCI (dyne·s/cm^5^) | 3138.1 | ±1262.78 | 3060.24 | ±1197.24 | 4049.43 | ±1613.08 | **<0.001** |
| CS (%) | 5.84 | ±2.66 | 5.88 | ±2.66 | 5.33 | ±2.59 | **0.043** |
| CC (mm^2^/kPa) | 0.65 | ±0.31 | 0.65 | ±0.31 | 0.55 | ±0.26 | **0.002** |
| DC (10^-3^/kPa) | 24.42 | ±12.66 | 24.92 | ±12.76 | 18.53 | ±9.69 | **<0.001** |
| YEM (10^3^/kPa) | 0.51 | ±0.36 | 0.49 | ±0.33 | 0.69 | ±0.58 | **<0.001** |
| β | 8.45 | ±4.86 | 8.24 | ±4.55 | 11.02 | ±7.20 | **<0.001** |
| Diameter (mm) | 6.3 | ±0.70 | 6.2 | ±0.70 | 6.6 | ±0.80 | **<0.001** |
| IMT (mm) | 0.75 | ±0.38 | 0.74 | ±0.36 | 0.86 | ±0.55 | **<0.001** |
| CIR^a^ | 3.48 | ±0.49 | 3.47 | ±0.49 | 3.67 | ±0.42 | **<0.001** |
| ^a^: transformed by using the natural logarithm. | | | | | | | |
| Numbers in bold letters indicate statistical significance | | | | | | | |
| β: Arterial stiffness index, CC: Compliance coefficient, CCI: Carotid characteristic Impedance, CIR: characteristic impedance ratio, CS: Circumferential strain, DC: Distensibility coefficient, IMT: Intima-media thickness, LDL: low-density lipoprotein-cholesterol, MAP: mean arterial pressure, MCI: mild cognitive impairment, PP: pulse pressure, SBP: systolic blood pressure, YEM: Young’s elastic modulus, Zc: aortic characteristic impedance | | | | | | | |

| Supplementary Table 3. Associations of indices of aortic and carotid stiffness with lower MMSE (MMSE<26): logistic regression | | | | | | | | | |
| --- | --- | --- | --- | --- | --- | --- | --- | --- | --- |
|  | Crude | | | Model 1 | | | Model 2 | | |
|  | OR | 95% CIs | | OR | 95% CIs | | OR | 95% CIs | |
| **Indices of carotid stiffness** | | | | | | | | | |
| CCI | **1.81** | **(1.55** | **-2.13)** | 1.23 | (0.97 | -1.57) | 1.24 | (0.97 | -1.58) |
| CS | **0.80** | **(0.64** | **-0.99)** | 0.95 | (0.74 | -1.22) | 0.96 | (0.75 | -1.23) |
| CC | **0.68** | **(0.54** | **-0.86)** | 0.78 | (0.58 | -1.04) | 0.78 | (0.58 | -1.05) |
| DC | **0.49** | **(0.37** | **-0.65)** | 0.81 | (0.58 | -1.12) | 0.82 | (0.59 | -1.13) |
| YEM | **1.44** | **(1.25** | **-1.67)** | **1.20** | **(1.01** | **-1.43)** | **1.19** | **(1.00** | **-1.43)** |
| β | **1.50** | **(1.29** | **-1.73)** | 1.17 | (0.97 | -1.41) | 1.17 | (0.97 | -1.40) |
| IMT | **1.48** | **(1.05** | **-2.08)** | 1.38 | (0.94 | -2.02) | 1.43 | (0.97 | -2.11) |
| **Index of aorta stiffness** | | | | | | | | | |
| Zc | **1.28** | **(1.08** | **-1.50)** | 1.06 | (0.86 | -1.30) | 1.06 | (0.86 | -1.31) |
| CIR^a^ | **1.48** | **(1.23** | **-1.79)** | 1.08 | (0.85 | -1.37) | 1.08 | (0.85 | -1.37) |
| odds ratios (per SD increase, with corresponding 95% CIs), numbers in bold letters indicate statistical significance. | | | | | | | | | |
| ^a^: transformed by using the natural logarithm. | | | | | | | | | |
| Model 1: Adjusted age, gender, education levels, MAP, HR, carotid diameter, height, weight | | | | | | | | | |
| Model 2: Adjusted age, gender, education levels, MAP, HR, carotid diameter, height weight, hypertension, DM, LDL, Smoking, | | | | | | | | | |
| β: Arterial stiffness index, CC: Compliance coefficient, CCI: Carotid characteristic Impedance, CIR: characteristic impedance ratio, CS: Circumferential strain, DC: Distensibility coefficient, IMT: Intima-media thickness, YEM: Young’s elastic modulus, Zc: aortic characteristic impedance | | | | | | | | | |

| Supplementary Table 4. Correlation matrix of carotid stiffness parameters. | | | | | | |
| --- | --- | --- | --- | --- | --- | --- |
|  | CCI | CS | CC | DC | YEM | β |
| CCI | 1 | -0.121* | -0.320* | -0.339* | 0.240* | 0.248* |
| CS | -0.121* | 1 | 0.798* | 0.865* | -0.637* | -0.726* |
| CC | -0.320* | 0.798* | 1 | 0.851* | -0.581* | -0.695* |
| DC | -0.339* | 0.865* | 0.851* | 1 | -0.656* | -0.729* |
| YEM | 0.240* | -0.637* | -0.581* | -0.656* | 1 | 0.866* |
| β | 0.248* | -0.726* | -0.695* | -0.729* | 0.866* | 1 |
| β: Arterial stiffness index, CC: Compliance coefficient, CCI: Carotid characteristic Impedance, CS: Circumferential strain, DC: Distensibility coefficient, YEM: Young’s elastic modulus | | | | | | |
| *:p value < 0.001 | | | | | | |
